# Investigating the Promoter of FAT10 Gene in HCC Patients

**DOI:** 10.3390/genes9070319

**Published:** 2018-06-26

**Authors:** Shuaichen Liu, Yu Jin, Dongwei Zhang, Jingbo Wang, Guangyi Wang, Caroline G. L. Lee

**Affiliations:** 1 Department of Hepatobiliary & Pancreas Surgery, The First Hospital, Jilin University, Changchun 130021, China; liuscmd@163.com; 2 Department of Biochemistry, National University of Singapore, Singapore 119077, Singapore; dongwei_z@hotmail.com (D.Z.); jingbowang1980@hotmail.com (J.W.); 3 Division of Medical Sciences, National Cancer Center, Singapore 169610, Singapore; jin.yu@nccs.com.sg; 4 Cancer and Stem Cell Biology Program, DUKE-NUS Graduate Medical School, Singapore 169857, Singapore

**Keywords:** FAT10, promoter, SNPs, expression, methylation

## Abstract

FAT10, which is also known as diubiquitin, has been implicated to play important roles in immune regulation and tumorigenesis. Its expression is up-regulated in the tumors of Hepatocellular Carcinoma (HCC) and other cancer patients. High levels of FAT10 in cells have been shown to result in increased mitotic non-disjunction and chromosome instability, leading to tumorigenesis. To evaluate whether the aberrant up-regulation of the FAT10 gene in the tumors of HCC patients is due to mutations or the aberrant methylation of CG dinucleotides at the FAT10 promoter, sequencing and methylation-specific sequencing of the promoter of FAT10 was performed. No mutations were found that could explain the differential expression of FAT10 between the tumor and non-tumorous tissues of HCC patients. However, six single nucleotide polymorphisms (SNPs), including one that has not been previously reported, were identified at the promoter of the FAT10 gene. Different haplotypes of these SNPs were found to significantly mediate different FAT10 promoter activities. Consistent with the experimental observation, differential FAT10 expression in the tumors of HCC patients carrying haplotype 1 was generally higher than those carrying haplotype II. Notably, the methylation status of this promoter was found to correlate with FAT10 expression levels. Hence, the aberrant overexpression of the FAT10 gene in the tumors of HCC patients is likely due to aberrant methylation, rather than mutations at the FAT10 promoter.

## 1. Introduction

FAT10, which is sometimes referred to as diubiquitin, was initially identified as one of the genes at the major histocompatibility complex locus in human chromosome 6 [1]. It is an 18 kDa protein and belongs to the ubiquitin-like modifiers (UBLs) family of proteins sharing 29% and 36% identity with ubiquitin at the N-terminus and C-terminus, respectively. FAT10 was suggested to function in a similar way as ubiquitin [2], in that it can act as a proteinaceous tag and target proteins for degradation by the 26S proteasome via attaching itself to that protein [3]. Three types of enzymes, namely E1, E2, and E3, are required for ubiquitination at three different steps. The similar process for FAT10 is called FAT10ylation, in which UBA6 and UBE3Z or USE1 act as the E1 and E2 enzyme. The E3 enzyme for FAT10 remains unknown [4,5]. Unlike ubiquitin, which is recycled from the degraded target proteins, FAT10 was reported to be degraded together with its target, resulting in a relatively short half-life [3]. Eukaryotic elongation factor 1A1 (eEF1A1) and UBE1 are examples for FAT10ylation, which will lead to their proteasomal degradation [6,7]. It was also found that the degradation of FAT10 can be further accelerated by its binding to the NEDD8 ultimate buster 1 long (NUB1L) protein [8], FAT10 forms a thioester with E1-L2, and E1-L2 is necessary for FAT10 conjugation in cells [9].

An in vivo study found that FAT10 ‘knock-out’ mice displayed minimal phenotypic changes. However, these mice are more sensitive to endotoxin challenge, and their lymphocytes are more susceptible to spontaneous apoptotic death [10]. Interestingly, a recent study reported that FAT10 knock-out mice have a longer overall lifespan and an elevated metabolic rate. There was a preferential use of fat for these mice, reduced circulating glucose and insulin levels, and an enhanced insulin sensitivity in metabolic tissues [11].

FAT10 has been implicated to play important roles in immune regulation and tumorigenesis. The cytokines IFN-γ and TNF-α were reported to up-regulate the expression of the FAT10 gene [1,12,13,14]. The FAT10 gene was also found to be up-regulated in the tumors of several cancers, including gastrointestinal and gynecological cancers [15]. In cells expressing high levels of the FAT10 protein [16] or induced by IFN-γ and TNF-α [14], increased mitotic non-disjunction and chromosome instability was observed, leading to tumorigenesis/malignancy [17]. Significantly, the interaction between FAT10 and MAD2 was found to be important for malignancy [17]. The drug silibinin was found to down-regulate FAT10, modulating IFN-γ/TNF-α-induced chromosome instability and sensitivity to apoptosis [18]. Interestingly, FAT10 was reported to modify and up-regulate the transcriptional activity of p53, the key guardian of the genome that plays an important role in tumorigenesis [19].

Genetic variation at the 5’UTR and coding region of the FAT10 gene was reported [20] to be associated with differential risk of Hepatocellular Carcinoma (HCC) in China. 

The expression of the FAT10 gene was reported to be regulated by p53 [21] during the cell cycle [22]. The role of FAT10 in tumorigenesis is thus implicated by the observation of an abnormally high expression of FAT10 in the tumors of several cancers [15], as well as increased mitotic non-disjunction and chromosome instability in cells expressing high levels of FAT10 [16]. There have also been findings that its expression is cell-cycle controlled [22] and positively regulated by TNF-α, a presumptive tumor promoter [3,23], but negatively regulated by p53 [21], the “*guardian-of-the-genome*” [24].

As the overexpression of FAT10 results in increased chromosome instability and plays a role in oncogenesis, elucidating the mechanism behind its aberrant expression in the tumors of HCC patients will facilitate the design of strategies to prevent its aberrant overexpression. We hypothesize that aberrant up-regulation of the FAT10 gene in HCC tumor tissues may be a result of mutations or aberrant methylation at the FAT10 promoter.

Mutations within the promoters of genes have been shown to result in the overexpression of several genes in cancer cells. For example, a mutation of the CDE/CHR (cell cycle-dependent element/cell cycle genes homology region) repressor elements at the survivin promoter was found to result in the overexpression of survivin in some cancer cells [25]. Similarly, mutations of the *hMSH*2 gene in suspected cases of hereditary nonpolyposis colorectal cancer (HNPCC) and sporadic early onset colorectal cancer patients were found to affect its promoter activity as well as the transcription start site and the transcriptional factor binding site, resulting in a novel DNA-protein complex [26]. Hotspot mutations in the Telomerase reverse transcriptase (TERT) promoter gene, occurring in 15% of patients with malignant pleural mesothelioma (MPM), was reported to up-regulate TERT in MPM [27].

Aberrant methylation at the promoters of genes may also account for the aberrant expression of the genes involved in the tumorigenesis process. The hypermethylation of CG (CpG) islands in the promoters of cancer-related genes is often associated with transcriptional inactivation [28,29,30]. Numerous publications demonstrated the involvement of DNA methylation in the silencing of tumor-suppressor genes [31,32,33,34,35]. Nonetheless, numerous other studies have also documented the reduced methylation of proto-oncogene promoter regions, suggesting a role for DNA hypomethylation in the activation of proto-oncogenes [36,37,38,39].

Since FAT10 is aberrantly overexpressed in the tumors of HCC and other cancer patients [15], this study aims to evaluate whether the dysregulation of FAT10 expression in the tumor tissues of HCC patients is due to mutations or aberrant methylation at the FAT10 promoter region 2. 

## 2. Materials and Methods

### 2.1. Human Samples

All of the human samples were obtained in accordance with the guidelines and the approval from the Institutional Review Board (IRB) of the National Cancer Centre of Singapore (NCCS) or the Singapore General Hospital (SingHealth CIRB 2006/442/B).

### 2.2. Study Design

**Figure 1:**
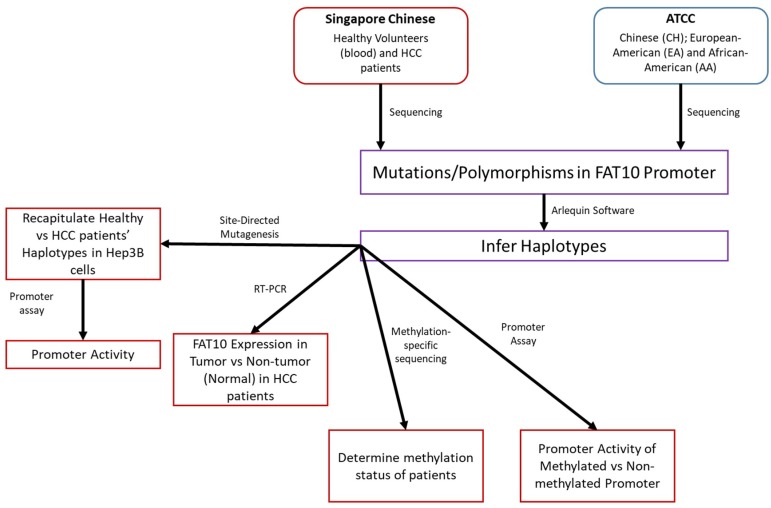
Study Design. Workflow of the project is presented, including the source of the patient samples and the techniques employed.

Figure 1 illustrates the design of this study.

### 2.3. Identification of Mutations/Polymorphisms at the FAT10 Gene Locus

A region of 1379 bp (−1066 upstream and +313 downstream of the transcriptional start site) of the FAT10 promoter was sequenced to screen for mutations or polymorphisms in the tumor and adjacent non-tumorous liver tissues from 37 HCC patients and 39 DNA samples from healthy people in the Singapore Chinese population with informed consent from the patients/volunteers and prior approval from the NCCS Institutional Review Board (SingHealth CIRB Ref 2006/442/B). This region of the promoter was selected, as it conferred the maximum promoter activity [21]. 

In addition, we sequenced the same region in the lymphoblastoid cell lines from three global populations, including 37 Chinese (CH), 31 European-American (EA), and 32 African-American (AA) from American Type Culture Collection (ATCC). Genomic DNA was isolated using the DNeasy^TM^ Tissue Kit (QIAGEN, Singapore). Primers for sequencing the 1379 bp fragment of the FAT10 promoter were designed according to the reference sequence AL031983 from Genbank using Primer Premier version 5.00 (Premier Biosoft International, Palo Alto, CA, USA). The primers used were F-5'ACTAATAGAGGTGGTTCCTTA (forward primer) and R-5′ CTCTCCCCAACTCTTGAAAGT (reverse primer). PCR reaction was carried out as follows: 95 °C for 15 min, then 30 cycles at 94 °C for 30 s, followed by 55 °C for 45 s and an extension at 72 °C for 1 min, followed by a final extension at 72 °C for 5 min. Sequencing was performed using the ABI PRI 3100 Genetic Analyzer (Applied Biosystems, Singapore). 

To identify somatic mutations, genetic variants of the tumor and adjacent non-tumorous liver tissues were compared. Polymorphisms were identified by comparing genetic variants in HCC patients versus healthy, Singaporean Chinese population controls. Haplotype frequencies were estimated using the program Arlequin [40], which is based on the expectation maximization (EM) algorithm [41]. 

**Figure 2:**
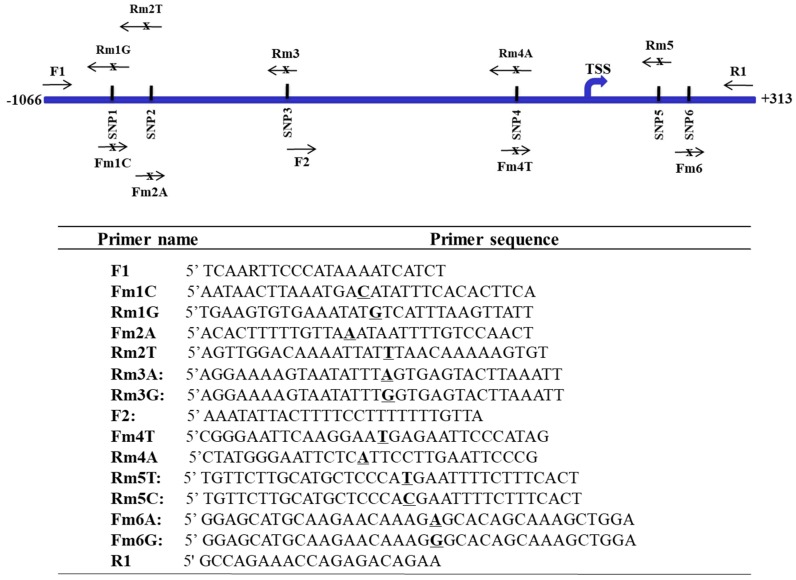
Primers for the generation of the different haplotypes of single nucleotide polymorphisms (SNPs) at the FAT10 promoter. Top: Schematic representation of the location of the various primers. Bottom: Table showing the sequence of the various primers. Bold underlined letters represent the SNP allele.

To evaluate the functionality of mutations/SNPs at the FAT10 promoter region, PCR site-directed mutagenesis as previously described [42] was employed to mutate the putative specific sites according to the haplotypes inferred for the HCC patients and non-HCC individuals using primers as shown in Figure 2. The PCR fragments with different mutated sites to represent the different haplotypes were inserted into a linearized vector where the various polymorphs of the FAT10 promoter drive the β-galactosidase (β-gal) reporter gene and the CMV promoter drives the enhanced green fluorescent protein (EGFP) gene to normalize for differences in promoter activity [21]. FAT10 promoter activity was analyzed as described previously [21]. All constructs were verified by sequencing to exclude PCR-induced nucleotide misincorporations prior to use.

### 2.4. Determination of FAT10 Gene Expression in HCC Patients

Total cellular RNA was isolated from 13 HCC patients using an RNeasy mini-kit (Qiagen). The quantitation of FAT10 and β-actin transcript levels was performed as previously reported [22]. Quantitative real-time PCR was performed on cDNA products using the RotorGene real-time PCR machine (Corbett Research, Sydney, Australia) and the QuantiTect SYBR Green RT-PCR kit (Qiagen). FAT10 and β-actin copy numbers were estimated from the threshold amplification cycle numbers using software supplied with the RotorGene real-time PCR machine. Results are expressed as relative FAT10 transcript levels normalized against β-actin transcript levels.

### 2.5. Determination of the Methylation Status at the FAT10 Promoter Using Methylation-Specific Sequencing

To determine the methylation status of the FAT10 promoter in HCC patients, genomic DNA from a tumor and an adjacent non-tumorous liver were treated with sodium bisulfite, as described [43]. All of the cytosine (C) in the genomic DNA would be converted to thymidine (T) by bisulfite treatment except for cytosines that are methylated. Primers were then designed according to the template whose C was converted to T. A half-nest PCR was utilized for this experiment because of the low amount of template. Primer F-sense-A 5’ TTATTTTTTGTGTTTGATAGTATGT with reverse primer FAT (+26)-R 5′ TCACATACTTCTCTCCTCAA were used in the initial PCR reaction, which comprises 95 °C for 15 min, followed by 25 cycles of 94 °C for 30 s, 55 °C for 45 s, and 72 °C for 50 s, followed by a final extension at 72 °C for 5 min. Thereafter, 5 μL of the initial PCR product was used as a template to perform a second round of PCR with forward primer F-sense-A 5’TTATTTTTTGTGTTTGATAGTATGT and reverse primer R-antisense-A 5’ ATCTTTATCTATTAAAACCACCTAA in a 50-μL reaction volume with the following PCR conditions: 95 °C for 15 min, followed by 30 cycles at 94 °C for 30 s, 53 °C for 45 s, and 72 °C for 45 s, followed by a final extension at 72 °C for 5 min. The PCR product was then sequenced. Kappa statistics [44] were then employed to determine if the methylation status correlates significantly with FAT10 expression.

### 2.6. Evaluation of the FAT10 Promoter Activity when the CG Nucleotides are Methylated

The previously described pFAT10-EGFP construct [21], in which the FAT10 promoter drives the β-galactosidase (β-gal) reporter gene while the constitutive cytomegalovirus (CMV) promoter drives the enhanced green fluorescent protein (EGFP) reporter gene, was utilized as a template to amplify the FAT10 promoter. The forward primer (5’-GTAAGGAGAAAATACAGCATCA-3’) was designed to anneal to the vector region immediately upstream of the FAT10 promoter, while the reverse primer (5’-AATTGGATCCGCCAGAAACCAGAGACAGAA-3’) anneals to the 3’ end of the FAT10 promoter, and contains the BamHI restriction enzyme site and a 4-bp (‘AATT’) sequence 5’ upstream of the BamHI site to facilitate more efficient restriction digestion. This region of the FAT10 promoter was then amplified using the Qiagen^®^ Multiplex PCR kit (Qiagen), according to the manufacturer’s instruction. The cycling condition was as follows: 95 °C for an initial 15 min followed by 25 cycles at 95 °C for 45 s, 55 °C for 45 s, and at 72 °C for 90 s, followed by a final extension at 72 °C for 10 min. The amplified product was purified using a QIAquick^®^ PCR Purification Kit (Qiagen). Half of the amplified product was treated with the CpG Methylase (M.SssI, New England Biolabs) and S-adenosylmethionine (SAM) to methylate the seven CpG sites at the FAT10 promoter, while the other half of the PCR product remained unmethylated or mock-methylated.

The EGFP reporter gene from the pEGFP-1 construct (Clontech) was also amplified using a forward primer (5’-CGCCTTCTCCAGGGATCCA-3’) that contains a BamHI site and anneals at the multiple cloning site upstream of the EGFP gene, and the reverse primer (5’-GAGTTTGGACAAACCACAACT-3’) that anneals to the 3’ end of the EGFP gene downstream of the poly A region of the EGFP gene. The amplification condition for the EGFP gene is similar to the amplification of the FAT10 promoter. The EGFP PCR product was also purified using the QIAquick^®^ PCR Purification Kit.

Both the methylated and unmethylated FAT10 promoter, as well as the EGFP amplified products, were digested with the BamHI restriction enzyme and purified using the QIAquick^®^ PCR Purification Kit. Ligation of the FAT10 promoter (methylated/unmethylated) with the EGFP reporter was then performed, and the ligated product was separated from the unligated product through gel electrophoresis. Only the ligated product was isolated and purified using the QIAquick^®^ Gel Extraction Kit (Qiagen) according to the manufacturer’s protocol.

To evaluate the activity of the methylated and unmethylated FAT10 promoters, the methylated/unmethylated FAT10 promoter-EGFP reporter fusion fragments were transfected into Hep3B cells using the siPORT™ Amine Transfection Agent (Ambion, Forster City, CA, USA) and the EGFP reporter activities evaluated.

### 2.7. Statistical Analysis

Fisher’s exact test was employed to evaluate the differences in allele or haplotype frequencies among the populations, as well as between the age-matched HCC and non-HCC individuals. The Student’s *t*-test was used to test the difference in the β-galactosidase activity between the constructs carrying the wild-type haplotype and other haplotypes in the FAT10 promoter. Similarly, Student’s *t*-test was also applied to identify the difference in promoter activity between the methylated and unmethylated groups.

## 3. Results

### 3.1. Only Polymorphisms, not Mutations, Were Identified in the ~1.3 kb Region of the FAT10 Promoter

To evaluate whether mutations/polymorphisms at the FAT10 promoter could account for the aberrant overexpression of FAT10 in the tumors of HCC patients, we sequenced approximately 1.3 kb of the FAT10 promoter in the tumor, and paired non-tumorous tissues from 37 Chinese HCC patients and 39 normal healthy Chinese individuals of a similar age. No difference in the sequence of the 1.3 kb of the FAT10 promoter examined was observed between the tumor tissues and adjacent non-tumorous tissues, suggesting that within the 1.3 kb region, there are no mutations that could account for the differences in the expression between the tumor and adjacent non-tumorous tissues.

Nonetheless, we identified six single nucleotide polymorphisms (SNPs) in this region (Table 1). All of these SNPs have been previously reported in the dbSNP database. Two of these SNPs are located in exon I, while the other four SNPs were found upstream of exon I. We examined the allele frequencies of all six SNPs in three populations, namely the Chinese (CH), European-Americans (EA), and African-Americans (AA), as well as Singapore Chinese HCC patients (HCC) compared with non-HCC Singapore Chinese individuals of similar age (non-HCC) (Table 1). Three SNPs (three, five, and six) had a high minor allele frequency of greater than 10% in at least one population, while the others were of low allele frequency (<5%) in all of the populations examined. The three major SNPs and low-frequency SNP4 (5’UR-169C > T) were observed in both the DNA of HCC samples and the age-matched non-HCC samples (Table 1). However, the low-frequency SNP1 was observed only in the HCC patients; SNP2 was not observed in either the HCC or age-matched non-HCC individuals in the local population (Table 1).

**Figure 3:**
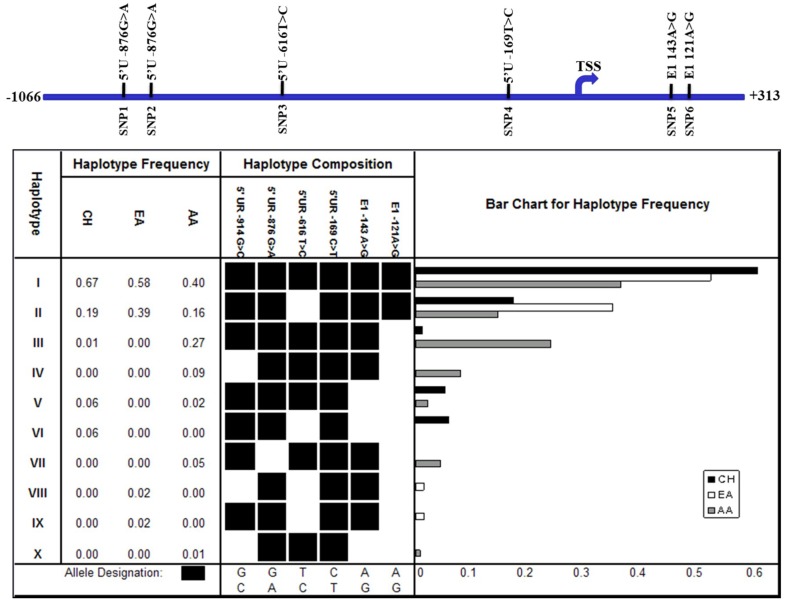
Haplotype of SNPs at the FAT10 promoter region in three ethnic populations. The six single nucleotide polymorphisms are schematically represented at the top panel. TSS: transcription start site. The frequencies of the haplotype of SNPs at the FAT10 promoter region as predicted in silico are shown at the lower panel. CH: Chinese, EA: European-American, and AA: African-American.

From the genotype data of these six SNPs, a total of 10 haplotypes were inferred from the different populations using an expectation maximization (EM) algorithm in the Arlequin^TM^ software program (Figure 3). Haplotypes I and II occurred at high frequencies in all of the populations examined, with haplotype I occurring at the highest frequency in the CH population, while haplotype II occurred at the highest frequency in the EA population (Figure 3). Haplotype III occurred at a relatively high frequency in the AA population (27%), but at a low frequency in the CH population (1%), and was not observed in the EA population. Haplotype V occurred at a low frequency in both the CH and AA populations, but was not observed in the EA population, while haplotypes IV, VII, and X were only observed at a low frequency in the AA (<10%) population, but not in the other populations. Haplotype VI only occurred at a low frequency in the CH population, while haplotypes VIII and IX were observed only in the EA population. These observations suggest that the profiles of haplotypes at the FAT10 promoter region differ in different populations. The profiles of the haplotypes of SNPs at the FAT10 promoter region between the HCC patients and non-HCC patients of similar age were also different, although the difference was not statistically significant (Fisher’s exact test, *p* > 0.05) (Figure 3. A total of eight haplotypes were inferred from the five SNPs observed in these samples, six of which (I, II, III, IV, V, and VII) were common to the haplotypes observed in the three populations (Figure 3). Four of the common haplotypes (I, II, III, and V) occurred in both the HCC and non-HCC individuals, although there were slight differences in the allele frequencies between the HCC and non-HCC individuals. Haplotypes IV, VII, and XI were only observed in the HCC patients, while haplotype XII was only observed in the non-HCC individuals.

**Figure 4:**
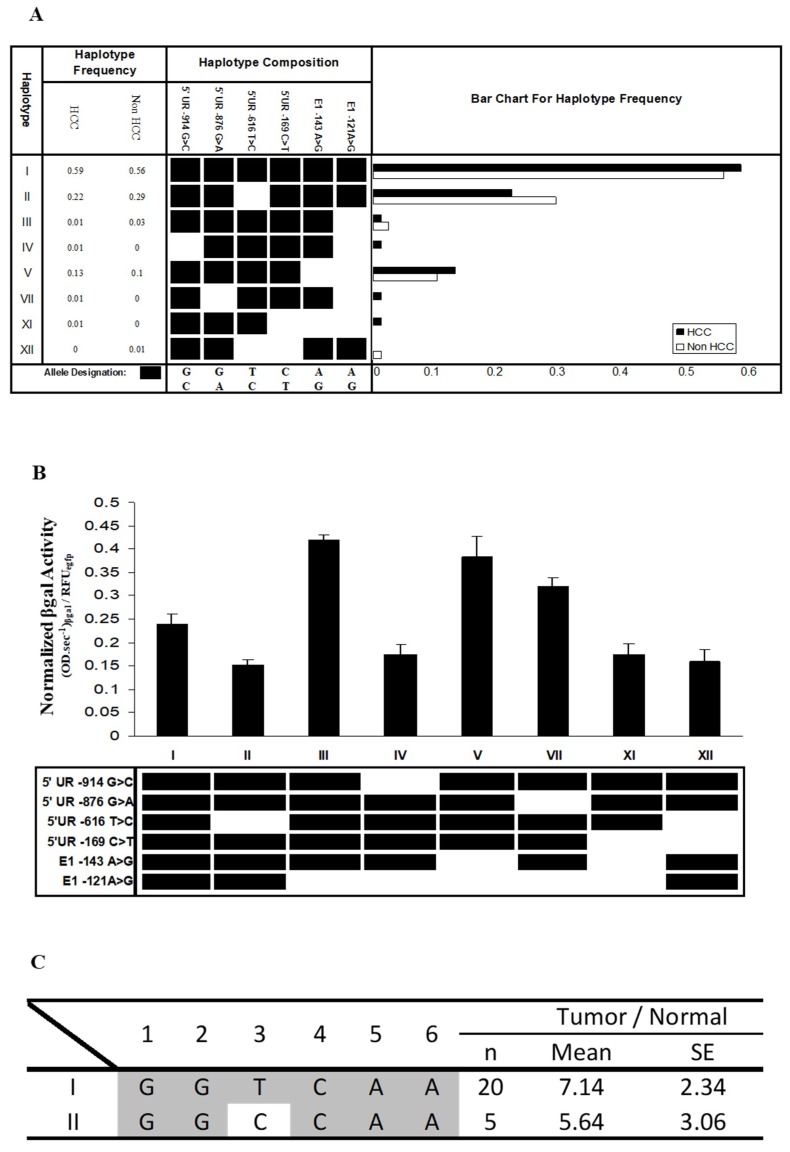
Haplotype of SNPs at the FAT10 promoter region in HCC patients and non-HCC individuals, and the FAT10 promoter activity mediated by the various haplotypes. (**A**) The frequencies of the haplotype of SNPs at the FAT10 promoter region, as predicted in silico. HCC: hepatocellular carcinoma patients, non-HCC: individuals of a similar age who have not been diagnosed with HCC; (**B**) Normalized β-galactosidase activity of the various FAT10 promoters carrying the different haplotypes in Hep3B cells. Data represent the mean and standard errors from four independent experiments. ** denotes significant difference (*p* < 0.01) between the various FAT10 promoter haplotypes and wild-type haplotype (I); (**C**) Fold difference in the normalized FAT10 transcript expression (as determined using reverse-transcription, real-time PCR) in the tumour versus non-tumorous liver tissues of HCC patients with haplotypes I and II.

Although no significant difference in the haplotype distribution was observed between HCC patients and normal individuals (Fisher’s exact test, *p* > 0.05) (Figure 4A), there were three low-frequency haplotypes that were only found in HCC patients (haplotypes IV, VII, and XI) and one (haplotype XII) that was only observed in normal individuals (Figure 4A). More samples may need to be examined before any conclusions can be drawn.

We proceeded to experimentally evaluate whether the SNPs at the FAT10 promoter may alter FAT10 promoter activity. The inferred haplotype of SNPs (Figure 4A) at the FAT10 promoter in HCC versus non-HCC patients of a similar age were recapitulated using PCR site-directed mutagenesis and cloned into the β-galactosidase reporter construct. The activity of the FAT10 promoter with the different haplotypes was then evaluated in Hep3B cells by analyzing the β-gal reporter gene activity after transfection. In this study, Hep3B cells, which do not express p53, were selected to facilitate our understanding of the role of the various haplotypes of polymorphisms on FAT10 promoter activity without the interaction of p53, which has been reported to modulate FAT10 gene expression/promoter activity [21]. As shown in Figure 4B, there are significant differences (*p* < 0.01) between the various mutated haplotypes and the wild-type haplotype GGTCAA. Interestingly, haplotypes III, V, and VII (GGTCAG, GGTCGG, and GATCAG) result in significantly higher FAT10 promoter activity, while haplotypes II, IV, XI, and XII (GGCCAA, CGTCAG, GGTTGG, and GGCTAA) mediate significantly lower FAT10 promoter activity. From these data, changing SNPs six, five, or two generally results in significantly higher FAT10 promoter activity. The only exception is when one or two of these are simultaneously changed with either one or four, as observed in haplotypes IV and XI. These haplotypes resulted in significantly lower FAT10 promoter activity. Changing SNPs three and four was observed to result in significantly lower FAT10 promoter activity. These results suggest that polymorphisms within the FAT10 promoter may alter FAT10 promoter activity and expression.

We proceeded to determine whether there is any association between the haplotype of SNPs and the differential expression of FAT10 in the tumors of HCC patients. Of the 37 patients that we genotyped, 20 displayed haplotype I, and were homozygous for the major allele of all six SNPs; meanwhile, five displayed haplotype II, in which SNP3 was the alternative C-allele. We did not examine the other patients, because there were heterozygous SNPs and the phase of the haplotype could not be determined with certainty. As evident in Figure 4C, although it was not statistically significant due to the small number of samples with the particular haplotype, the ratios of FAT10 expression in the tumor and non-tumor tissues of patients with haplotype II (5.64 ± 3.06) was generally lower than those carrying haplotype I (7.14 ± 2.34). This observation is consistent with the FAT10 promoter reporter assay, as shown in Figure 4B. A possible explanation for this observation is that the change from the T to the C allele in SNP3 between haplotypes I and II abolished the binding of the myocyte enhancer factor, resulting in a decrease in the expression of the gene. These data suggest that the different haplotypes of the SNPs affected the differential FAT10 expression in the tumors of the HCC patients. It would be interesting to determine whether the different haplotypes of the SNPs might influence the prognosis of the HCC patients.

### 3.2. Differential Methylation at the FAT10 Promoter Was Observed between Tumor and Adjacent Normal Liver Tissues of HCC Patients

**Figure 5:**
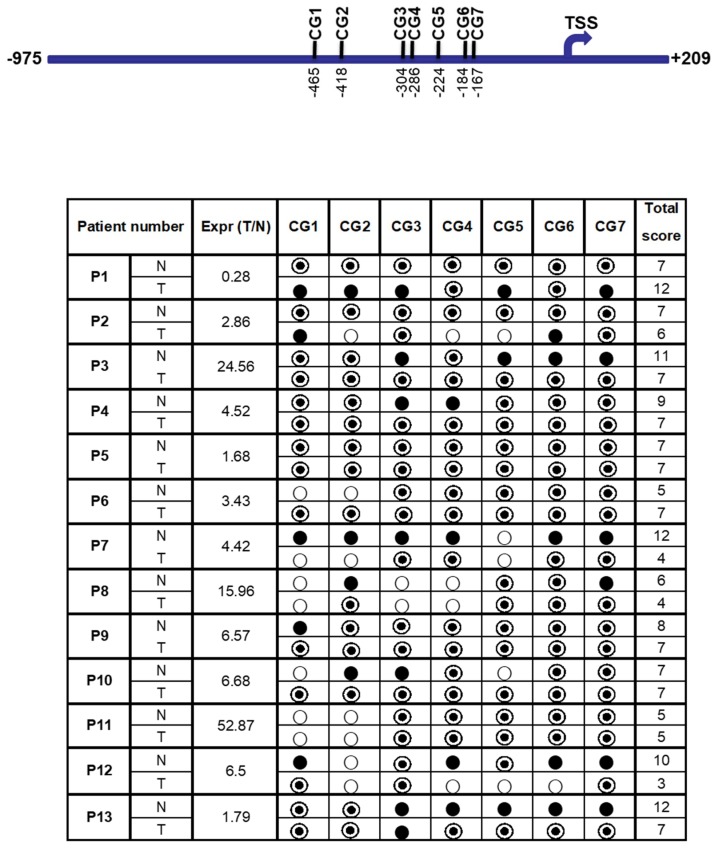
Correlation between the methylation status at the FAT10 promoter and FAT10 transcript expression in HCC patient tissues. The seven CG dinucleotides are schematically represented at the top panel. TSS: Transcription Start Site. The bottom panel shows the correlation between the methylation status and the fold difference in FAT10 transcription expression between the tumor and non-tumorous liver tissue of each HCC patient. The last column shows the sum of the scores of either the fully methylated (score = 2) denoted by ●, half methylated (score = 1) denoted by ◎, and unmethylated (score = 0) denoted by ○.

A total of seven CG dinucleotides (CG-1 to CG-7) reside in the region from −975 to +209 bp of the FAT10 promoter, which showed the highest promoter activity (Figure 5). All of these CG dinucleotides reside upstream of the TSS (transcription start site). In order to evaluate the effect of DNA methylation on FAT10 expression, methylation-specific sequencing was utilized to evaluate the methylation status of different CG dinucleotides in the FAT10 promoter region.

Tumor and adjacent non-tumorous liver tissue samples from 13 HCC patients were used in this study. As evident in Figure 5, except for patients 6, 10, and 11, the methylation status of the other 10 HCC patients generally correlated with their transcript expression levels. Less methylation in the tumor tissues is correlated with the higher expression of FAT10 in the tumor tissues (patients 2, 3, 4, 7, 8, 9, 12, and 13). Conversely, as evident in patient 1, a higher methylation in the HCC tumor is correlated with lower FAT10 transcript expression. Likewise, no significant differential methylation was observed in patient 5, in whom FAT10 was not found to be differentially expressed in the tumors compared with the adjacent normal tissues. Utilizing kappa statistics [44], the methylation status was found to inversely correlate significantly with FAT10 expression (κ value = 0.628).

**Figure 6:**
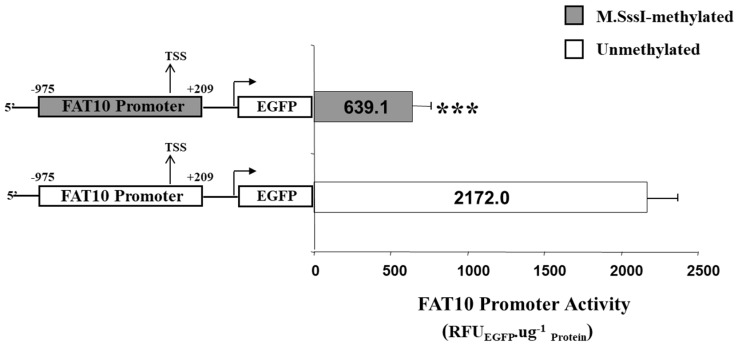
Activity of the FAT10 promoter that has been methylated in vitro versus the promoter that has not been methylated. The FAT10 promoter that has either been methylated with M.SssI or mock methylated (unmethylated) is fused with the enhanced green fluorescent protein (EGFP) reporter protein and transfected into Hep3B cells. The FAT10 promoter activity expressed through EGFP protein levels is determined. Figure shows the mean and standard error from four (methylated) and five (unmethylated) independent experiments. *** denotes a significant difference (*p* < 0.001) in activity between the unmethylated and methylated FAT10 promoter.

To experimentally demonstrate whether the methylation of the CG dinucleotides will result in lower FAT10 promoter activity, the entire FAT10 promoter region was methylated in vitro using SssI methylase (M.SssI, New England Biolabs) and ligated upstream of the EGFP gene. The FAT10 promoter (methylated/unmethylated)-EGFP fusion was then transfected into the Hep3B cells, and the FAT10 promoter activity was evaluated by quantitating the EGFP protein levels. As evident in Figure 6, the methylated FAT10 promoter mediated a significantly lower (>three-fold) reduction in EGFP reporter activity compared with the unmethylated promoter.

Hence, methylation may play a role in the regulation of the FAT10 expression level.

## 4. Discussion

### 4.1. No Mutations at the FAT10 Promoter Were Observed

The overexpression of FAT10 has been observed in the tumors versus the adjacent non-tumorous tissues of HCC and gastrointestinal and gynecological cancers [15]. The high level of the FAT10 protein in cells was reported to increase mitotic non-disjunction and chromosome instability [16], leading to tumorigenesis/malignancy [17] through the interaction of FAT10 with the mitotic checkpoint protein, MAD2 [17]. Therefore, one of the main objectives of this study was to elucidate the mechanism behind the aberrant expression of FAT10 in the tumor tissues of HCC patients. Since mutations found in the promoter region had been correlated with the overexpression of genes in cancer cells [25,45], we sequenced ~1.3 kb of the FAT10 promoter region from a tumor and the adjacent non-tumorous liver tissues of 37 HCC patients to screen for mutations. However, no mutations were identified within the 1.3-kb region of the FAT10 promoter, suggesting that the difference in FAT10 expression levels between the HCC tumor and adjacent non-tumorous tissues cannot be accounted for by mutations within the 1.3-kb region of the FAT10 promoter. However, this process did not rule out mutations upstream of this region, accounting for the differential expression between the tumor and adjacent non-tumorous tissues.

Nonetheless, although no mutations were found in the tumor of HCC patients that may account for differential FAT10 expression between the tumor and the adjacent non-tumorous tissues, we identified polymorphisms in both the tumor as well as the adjacent non-tumorous tissues of the HCC patients. As these polymorphisms were found in both the tumor and the adjacent non-tumorous tissues, they are unlikely to account for the differential FAT10 expression that was observed. Nevertheless, these SNPs may affect the basal FAT10 expression and account for the differences in the basal FAT10 expression among different individuals. 

Polymorphisms in the promoters of genes have previously been demonstrated to affect promoter activity and hence gene expression [46,47,48,49]. Since high FAT10 has been associated with increased chromosomal instability [16], and chromosomal instability is one of the hallmarks of cancer, it raises the possibility that individuals with polymorphisms that result in high FAT10 promoter activity and expression may have higher risks of having cells with unstable chromosome numbers, leading to a higher risk of developing cancer.

A total of six polymorphisms were identified. Two of the six SNPs were found to be monomorphic in normal individuals, and occur at a low frequency in the HCC patients. Polymorphisms within the FAT10 gene locus were reported [20] to be associated with a risk of HCC in Chinese patients. This Chinese study primarily focused on polymorphisms at the 5’ UTR, coding, and 3’ UTR regions of the gene, and did not examine most of the polymorphisms at the FAT10 promoter region, except for two SNPs (rs362535, rs2272991) at the 5’ UTR region. While they reported that the alternative alleles of these two SNPs were significantly associated with decreased HCC risk, our data did not show any significant difference. Further study with a larger cohort may be necessary to resolve these different observations. 

While seven haplotypes can be inferred from the six SNPs identified in the HCC patients, only five haplotypes can be inferred from the five SNPs that were found in the normal individuals (Figure 4A). Three of the seven haplotypes are found exclusively in HCC patients, while one haplotype exists only in normal individuals (Figure 4A). Despite these differences, Fisher’s exact test showed no statistically significant differences between the SNPs or haplotypes of the HCC patients and normal individuals (*p* > 0.05). As those SNPs and haplotypes that are different between the HCC patients and normal individuals occur at very low frequency, more samples need to be examined before a conclusion can be made. The haplotypes of the SNPs in this study cannot be compared with those from the study by Yuan et al. [20], since their study focused on haplotypes from exonic SNPs, while our study focused on haplotypes from promoter SNPs.

From in silico analyses, all of the polymorphisms observed at the FAT10 promoter either remove an existing putative transcription factor binding site, introduce a new putative transcription factor binding site, or change a putative binding site to another (Table 1). Experimentally, we demonstrated that the different haplotypes of FAT10 polymorphisms resulted in significantly different FAT10 promoter activity compared with the wild-type haplotypes (Figure 4B). Interestingly, it seems that changing SNP3 and/or SNP4 generally resulted in lower FAT10 promoter activity, while changing SNP2, SNP5, and/or SNP6 generally resulted in higher FAT10 promoter activity. The exceptions are when these SNPs are changed simultaneously with either SNP1 or SNP4 (haplotype IV and XI), which then resulted in significantly lower FAT10 promoter activity. Although significant, the changes observed with the different haplotypes was less than twofold, unlike the differential expression observed between the HCC tumor and adjacent non-tumorous tissues, which could be more than five-fold. Consistent with the promoter reporter assays, we also observed that the differential FAT10 expression in the tumors of HCC patients with haplotype I is slightly higher (7.14 ± 2.34) than the tumors of HCC patients with haplotype II (5.64 ± 3.06). These results suggest that although individuals with different FAT10 promoter haplotypes may have different basal levels of FAT10 expression, these differences may not be large enough to modify the risk of the individuals to develop HCC or have cells with different chromosome stability potential. This is consistent with our observation of no statistically significant differences between the SNPs or haplotypes of the HCC patients and normal individuals (*p* > 0.05).

### 4.2. Differential Methylation May Account for the Differences in FAT10 Gene Expression between HCC Tumor and Adjacent Non-Tumorous Tissues

CG dinucleotides are present in the regulatory regions of many genes [50]. In normal cells, the cytosines in the CG dinucleotides generally remain unmethylated [50]. However, in the promoter sequences of genes associated with certain cancers or inherited diseases, more CG dinucleotides at the promoter region were found to be methylated [51]. The methylation status in the DNA of humans and other mammals plays an important role in determining whether some genes are expressed or not. Abnormal DNA methylation plays an important role in other developmental diseases as well. Abnormal increases or decreases in DNA methylation are often observed in human cancers, and may contribute to their development [28,51].

Seven CG sites were observed to reside in the FAT10 promoter region. In order to study the correlation between the methylation status of these CG dinucleotides and the aberrant expression of FAT10 in HCC patients [15], we performed methylation-specific sequencing to screen the methylation status of these CG dinucleotides at the FAT10 promoter in 13 HCC patients. We found that generally, higher FAT10 expression is correlated significantly with reduced methylation of the CGs at the FAT10 promoter (κ value = 0.628) (Figure 5). The exceptions were patients 6, 10, and 11, whereby although the HCC tumor tissues had higher FAT10 expression, the methylation status of the tumor was either more in the tumor (P6), or was no different from the adjacent normal tissues (P10 and 11). It is possible that the differential methylation of other unexamined sites correlates with the FAT10 expression in these patients. Nonetheless, the in vitro methylation of this region of the FAT10 promoter results in lower FAT10 promoter activity (Figure 6), which is consistent with the above observations.

Curiously, although the promoters of most genes are generally hypomethylated in adult tissues, the adjacent non-tumorous tissues of HCC patients seem to display hypermethylation at the FAT10 promoter, while the tumorous tissues seem to be hypomethylated. One possible explanation for this observation is that the adjacent non-tumorous tissues of HCC are generally cirrhotic, and cirrhosis may have resulted in the hypermethylation of the FAT10 promoter. The progression from a cirrhotic liver to a tumorous liver may then change the methylation status of the FAT10 promoter to activate the FAT10 gene. This hypothesis remains to be examined. 

Future studies could validate these observations in additional cell lines, as well as additional healthy volunteers/HCC patient samples, and determine the contribution of non-genetic factors. It would also be pertinent to address whether the polymorphisms or differential methylation at the FAT10 promoter would modulate the response of the FAT10 promoter to inflammatory cytokines, including TNFα or IFNγ. As the expression of the FAT10 gene was reported to be regulated by p53 [21], it would also be interesting to evaluate whether p53 differentially modulates FAT10 expression in cells with different haplotypes of polymorphisms, or a different methylation status at its promoter. Importantly, the association between the FAT10 promoter and HCC pathogenesis can be further clarified, and the role of DNA methyltransferases in modulating the promoter activity of FAT10 can be elucidated. 

## 5. Conclusions

In summary, although no mutations were identified at the FAT10 promoter in the tumor of HCC patients, polymorphisms at this promoter was identified, which mediated differential FAT10 promoter activities. Notably, the methylation status at this promoter correlated significantly with FAT10 expression levels as well as differential promoter activity. Thus, epigenetics (methylation) play an important in regulating the expression of FAT10. 

## Figures and Tables

**Table 1:** Profile of allele frequency of single nucleotide polymorphisms (SNPs) at the FAT10 promoter.

No.	ID	SNP Name	Transcription Factor Binding Sites		Population		n	Allele Frequency (%)		Pairwise Differences Fisher‘s Exact *p*-Value				
1	rs11962004	5'UR -914 G>C	**G**	**C**				**G**	**C**	**CH**	**EA**	**AA**	**Age-matched**	
													**non-HCC**	**HCC**
				Octamer-binding factor 1		CH	37	98.6	1.4		1.0	4.9 × 10^−2^	0.5	
						EA	31	98.4	1.6			0.1		
				TCF11/KCR-F1/Nrf1		AA	32	90.6	9.4					
				homodimers	Age-matched	nonHCC	39	100.0	0.0					1.0
						HCC	56	99.1	0.9					
2	rs115899746	5'UR -876 G>A	**G**	**A**				**G**	**A**	**CH**	**EA**	**AA**	**Age-matched**	
													**non-HCC**	**HCC**
			GATA-binding factor 2			CH	37	100.0	0.0		1.0	0.1	1.0	
						EA	31	100.0	0.0			0.2		
			Hepatic nuclear factor 1			AA	32	95.3	4.7					
					Age-matched	nonHCC	39	100.0	0.0					1.0
						HCC	56	99.1	0.9					
3	rs362513	5'UR -616 T>C	**T**	**C**				**T**	**C**	**CH**	**EA**	**AA**	**Age-matched**	
													**non-HCC**	**HCC**
						CH	37	74.3	25.7		0.1	0.2	0.6	
			Myocyte enhancer			EA	31	58.1	41.9			1.5 × 10^−0.3^		
			factor			AA	32	84.4	15.6					
					Age-matched	nonHCC	39	69.2	30.8					0.6
						HCC	56	73.2	26.8					
4	rs189072824	5'UR -169 C>T	**C**	**T**				**C**	**T**	**CH**	**EA**	**AA**	**Age-matched**	
													**non-HCC**	**HCC**
				HMG box-containing protein 1		CH	37	74.3	25.7		1.0	1.0	1.0	
				TEF-1 related muscle factor		EA	31	58.1	41.9			1.0		
			HMGI(Y)			AA	32	84.4	15.6					
				POU-factor Tst/Oct-6	Age-matched	nonHCC	39	98.7	1.3					1.0
				Octamer-binding factor 1		HCC	56	99.1	0.9					
5	rs362535	e1 82 A>G	**A**	**G**				**A**	**G**	**CH**	**EA**	**AA**	**Age-matched**	
													**non-HCC**	**HCC**
				Egr-1/Krox-24/NGFI-A		CH	37	87.8	12.2		3.9E-03	0.1	0.8	
			Brn-2, POU-III protein			EA	31	100.0	0.0			0.1		
			class	RBP-Jkappa/CBF1		AA	31	96.8	3.2			0.5		
					Age-matched	nonHCC	39	89.7	10.3					0.3
						HCC	56	83.9	16.1					
6	rs2272991	e1 104 A>G	**A**	**G**				**G**	**C**	**CH**	**EA**	**AA**	**Age-matched**	
													**non-HCC**	**HCC**
				PPAR/RXR heterodimers		CH	37	86.5	13.5		0.1	1.0 × 10^−4^	1.0	
						EA	31	96.8	3.2			3.3 × 10^−0.8^		
				Gut-enriched Krueppel-like		AA	32	56.3	43.8					
				factor	Age-matched	nonHCC	39	87.2	12.8					0.5
						HCC	56	83.0	17.0					
